# Dynamical observations on the crack tip zone and stress corrosion of two-dimensional MoS_2_

**DOI:** 10.1038/ncomms14116

**Published:** 2017-01-18

**Authors:** Thuc Hue Ly, Jiong Zhao, Magdalena Ola Cichocka, Lain-Jong Li, Young Hee Lee

**Affiliations:** 1 IBS Center for Integrated Nanostructure Physics (CINAP), Institute for Basic Science, Sungkyunkwan University, Suwon 440-746, Korea; 2 Department of Energy Science, Sungkyunkwan University, Suwon 440-746, Korea; 3 Physical Sciences and Engineering, King Abdullah University of Science and Technology, Thuwal 23955-6900, Saudi Arabia

## Abstract

Whether and how fracture mechanics needs to be modified for small length scales and in systems of reduced dimensionality remains an open debate. Here, employing *in situ* transmission electron microscopy, atomic structures and dislocation dynamics in the crack tip zone of a propagating crack in two-dimensional (2D) monolayer MoS_2_ membrane are observed, and atom-to-atom displacement mapping is obtained. The electron beam is used to initiate the crack; during *in situ* observation of crack propagation the electron beam effect is minimized. The observed high-frequency emission of dislocations is beyond previous understanding of the fracture of brittle MoS_2_. Strain analysis reveals dislocation emission to be closely associated with the crack propagation path in nanoscale. The critical crack tip plastic zone size of nearly perfect 2D MoS_2_ is between 2 and 5 nm, although it can grow to 10 nm under corrosive conditions such as ultraviolet light exposure, showing enhanced dislocation activity via defect generation.

## Abstract (web summary)

How well the linear elastic fracture picture holds at small length scales and systems with reduced dimensionality remains an active area of inquiry. Here authors use *in situ* electron microscopy to study fracture in MoS_2_ monolayers and report dislocation emission rates greater than expected accompanying crack propagation.

Fracture is a process relevant to many kinds of applications of materials. Competition between two modes, namely, the crystal plane cleavage and dislocation nucleation at the crack tip, is the main factor governing the ductile or brittle behaviour of crystals^1^,^2^. Since the establishment of the well-known Griffith model^3^, it has been questioned whether linear elastic fracture mechanics hold true in the crack tip down to all length scales and, specifically, whether it holds true for recently developed two-dimensional (2D) materials^4^,^5^ is debatable.

Direct electron microscopy observations of crack tips in ceramic materials such as Si, SiC and Al_2_O_3_ show perfect fringe lattices at the vicinity of the crack tips, and confirmed the absence of dislocations^6^. The revealed atomic sharpness at the crack tip without any blunting process is a signature of pure brittleness. Recently, 2D materials such as graphene and transition metal dichalcogenide (TMD) come into view for the realization in fabrication and for their exceptional physical properties^7^. In particular, in the monolayer form, they only consist of one atom (such as graphene) to three atoms (such as TMD) in thickness. The basal plane (intralayer) fracture of the bulk type covalent-bonding graphite or 2h-molybdenite (hexagonal MoS_2_, to differentiate from the tetragonal phase) is purely brittle at room temperature^8^, and their fracture toughness G_IC_ equals to 2*γ*, where *γ* is the surface energy per unit area^8^. Indentation tests using atomic force microscopy on monolayer graphene^4^,^9^ and monolayer hexagonal(h)-MoS_2_ (ref. 5) have demonstrated ultra-high strength that is close to the theoretical limit, followed by catastrophic failure, and transmission electron microscopy (TEM) could distinguish the transgranular fracture from intragranular fracture; however, no atomistic information was given^9^. The fracture toughness of monolayer graphene was quantitatively measured by *in situ* TEM with supportive molecular dynamics simulations, suggesting pure brittleness^10^. The cracks in monolayer graphene are along the preferable zigzag edge^11^. Meanwhile, several types of basal plane dislocations, or ‘zero-dimensional' dislocations, can emerge and are mobile in graphene or TMD^12^,^13^,^14^, especially at high temperature or under electron beam irradiation, indicative of the easier dislocation dynamics in 2D form than in bulk.

It is expected that the defect-free nature and high strength in 2D^4^,^5^ may provide a higher chance to achieve the onset of plasticity, but on the other hand, most molecular dynamics simulations show that the fracture of graphene as well as MoS_2_ is brittle^15^,^16^. To understand the atomistic features when fracture occurs in 2D materials, a direct observation of the cracking process at the crack tip is necessary from the viewpoint of basic physics and practical use. Previous work by *in situ* TEM demonstrated the capability of dynamical atomic-scale observations on the sublimation and cracking process of few layer graphene^17^ or monolayer graphene^17^. Moreover, environment-assisted cracking in MoS_2_ also needs to be investigated and compared with pure cracking that is of particular importance in real-world applications. The environmental susceptibility of MoS_2_ has drawn much attention^18^ that would have significant influence on their physical properties^19^.

Here, by using *in situ* transmission electron microscopy to resolve the atomic structures, we directly observed the limited plastic behaviour at the forefront of a singular crack tip of monolayer h-MoS_2_. The cracks can be initialized at holes, and electron beam damage and especially creation of sulfur vacancies was carefully suppressed to ensure the crack propagation is close to pure mechanical loading. The emission, glide and climb of the dislocations that can influence the propagation of the cracks were captured. High-quality MoS_2_ monolayer samples with 1% sulfur deficiencies have around 2–5 nm size plastic zone (dislocation distributed area) near the crack tip in vacuum and extends to 5–10 nm under corrosive environments. Our results also suggest that the specific 2D geometry improves ductility, in combination with the reported ultrahigh strength of 2D graphene^4^ or MoS_2_ (ref. 5), creating potential applications for these materials.

## Results

### Sample preparation and introduction of cracks

The monolayer MoS_2_ samples were grown on sapphire using chemical vapour deposition^20^, and then transferred to TEM grids using the polymethyl methacrylate (PMMA) method as a free-standing sheet^21^. Excellent quality of the as-grown MoS_2_ crystal was confirmed by TEM (Supplementary Fig. 1), revealing the sulfur deficiencies lower than 1% (Supplementary Table 1). Owing to the high specific surface area, it was inevitable that some organic adsorbents remained on the sample surface after transfer (Supplementary Fig. 2). Absorbents on the MoS_2_ surface can be activated and evaporated gradually by electron beam irradiation. From a mechanical point of view, these loosely contacting adsorbents can stabilize the 2D samples in the cracking process that we will discuss later. The vacuum in the JEOL ARM-200F TEM is >2 × 10^−5^ Pa and all TEM measurements were conducted at room temperature under 80 kV. During the *in situ* observation of cracking, the electron beam was kept sufficiently low or entirely turned off and only turned on (beam intensity 0.01 pA nm^−2^) for taking images with a short exposure time (0.32 s; see Supplementary Fig. 1 and Supplementary Table 1 for more defect analysis in MoS_2_ in all experimental conditions). The occurrence of the beam damage on MoS_2_ requires much higher beam intensity (Supplementary Fig. 1)^22^.

**Figure 1:**
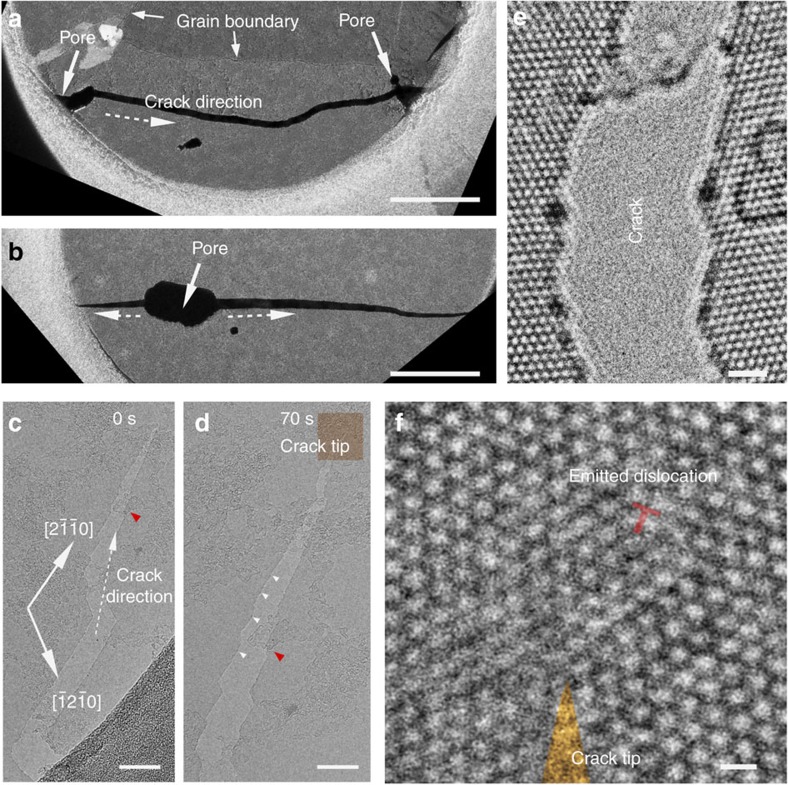
**TEM observations of cracks of monolayer MoS**_**2**_. (**a**) Dark-field image of one crack initiated from the left pore through the entire hole, finally reaching the right pore, avoiding the grain boundary. Scale bar, 200 nm. (**b**) A similar crack initiated from the centre pore. Scale bar, 200 nm. (**c**) Bright-field image at the higher magnification reveals the microscopic cracking path. Scale bar, 45 nm. (**d**) The same crack as (**a**) after 70 s and several steps on the post crack surface are highlighted. The red triangles in **c**,**d** mark the same area on the sample. Scale bar, 30 nm. (**e**) HRTEM image for a cracked surface. Scale bar, 1 nm. (**f**) HRTEM image for an emitted dislocation close to a crack tip. Scale bar, 300 pm.

According to the criterion of continuum fracture mechanics, a crack starts to grow when the maximum stress intensity (*K*) on the sample exceeds the critical stress intensity factor (*K*_c_, fracture toughness). We confirmed that crack propagation is preferably initiated at some pores (either in pristine samples or newly created by condensed electron beam; see dark-field (DF) TEM images in Fig. 1a,b) that is congruent with the fact that the stress intensity factor is proportional to the square root of the dimension of defects^3^. The cracks can quickly run through the entire sampling area (suspended on 1 μm holes of the TEM grid, Fig.1a,b), or can stop in the centre because of relaxation of stresses and stabilization by adsorbents (Supplementary Fig. 3). We also note that the crack does not usually follow the grain boundaries (Fig. 1a), similar to previous findings^8^. In the following part, our discussion will be restricted to intragranular cracking.

Figure 1c,d presents two snapshots of cracking during 70 s. Generally, straight cracking surfaces can be seen, whereas the cleavage plane of monolayer MoS_2_ is determined by diffraction as the close-packed 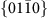 planes or a zigzag edge. The high-resolution transmission electron microscopy (HRTEM) image of the post cracking surfaces shows the two opposite S-terminated edge and Mo-terminated edge by direct decohesion (Supplementary Figure 4). Owing to the tensile stress direction not being exactly perpendicular to any cleavage plane, the nominal cracking direction is between [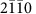] and [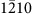] (Fig. 1c–e, and Supplementary Fig. 5). In addition to these proofs of brittleness, we also found some irregular steps or defective sites (highlighted by white triangles in Fig. 1d) on the crack surfaces. More importantly, dislocations that are absent in the pristine sample can be seen by HRTEM close to the crack tip (Fig. 1f). This dislocation structure consists of 4–6 cores with Burgers vector 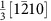 (Supplementary Fig. 6), and dislocation cores in MoS_2_ may have more types^23^. These phenomena suggest that there might be other mechanisms accounting for the 2D MoS_2_ cracking apart from decohesion.

### *In situ* observation of fracture

**Figure 2:**
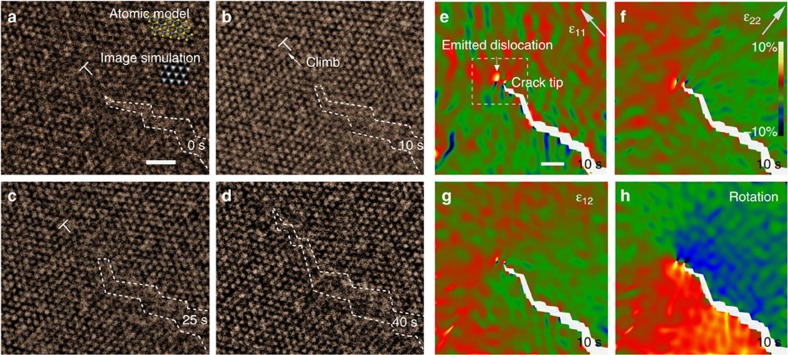
***In situ*** **TEM series revealing crack propagation and dislocation climb**. (**a**–**d**) The *in situ* TEM image series showing a crack propagation at 0, 10, 25 and 40 s. All the images are aligned to show the same area (drift compensation) by aligning far-field less strain zones. Cracks are highlighted within the white-dashed lines in the lower right corners. The atomic models for perfect MoS_2_ and multislice TEM image simulations of the atomic model corresponding to experimental TEM conditions are shown in the inset of **a**. The electron beam was turned off during the cracking process but only turned on to take images (exposure time 0.32 s), to exclude additional electron beam effect on the cracking. There is one edge dislocation at 0 s, highlighted by ‘T'. It starts to climb because of the stress of the crack tip at 10 s, and disappears at 40 s when the crack extends further. Scale bar (**a**), 1 nm. (**e**,**f**) The GPA strain analysis of the 10 s image. The box in **e** highlights the corresponding area shown in the HRTEM images in **a–d**. Axial strains with directions marked in the upper right corners in **e**,**f**, shear strain shown in **g** and rotation shown in **h**. Scale bar (**e**), 5 nm.

Figure 2a–d presents one case of *in situ* cracking during 40 s by HRTEM images. There is one dislocation (labelled by ‘T') near the crack tip. After 10 s, the dislocation climbs along the cracking direction by 1 nm. The geometric phase analysis (GPA)^24^ was carried out on the original TEM images at 10 s, and the axial strains, shear strain and lattice rotation are presented in Fig. 2e–h. The dislocation at the crack tip is highlighted in Fig. 2e. The distribution of the tensile strain ɛ_22_ at the crack tip zone in Fig. 2f resembles the tensile strain distribution for normal Mode I cracking^25^. However, the shear strain also exists (ɛ_12_ in Fig. 2f). Meanwhile, the local strain field near the crack tip is not uniform but contains a lot of high-frequency fluctuations (Fig. 2e–g) that can be attributed to the easy three-dimensional (3D) fluctuations of 2D materials^13^ when subjected to complex stress field near the crack tip. Because of the coexistence of tensile, shear and tear stress, the crack of MoS_2_ here should be a mixed mode (I, II and III) cracking^25^. At the crack tip, because of the large strain gradient in the crack tip zone, the two parts of the monolayer MoS_2_ crystals on the two sides of the dislocations can have some angle difference that can be locally considered as 2 nm-sized grains.

**Figure 3:**
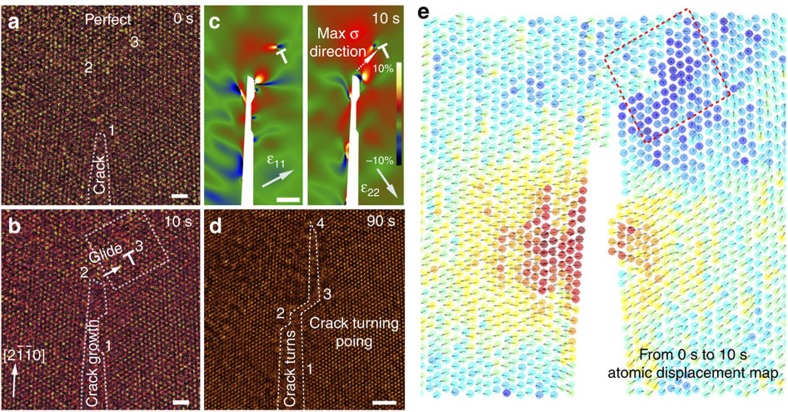
**The** ***in situ*** **TEM observation of dislocation emission and crack turning**. (**a**,**b**) The *in situ* TEM observation of cracking at 0 and 10 s. Electron beam is off during cracking and only turned on for imaging. The series images are spatially aligned (drift compensation). There is no dislocation in view at 0 s, whereas one fresh dislocation emerges in the box at 10 s, highlighted by ‘T'. Scale bars, 1 nm. (**c**) The GPA strain analysis for the axial strains at 10 s. Scale bar, 2 nm. (**d**) TEM image showing the crack turn after 90 s. All the related positions on the samples are labelled as 1, 2, 3 and 4 in **a**,**b**,**d**. The maximum tensile stress direction in **c** is in agreement to the following crack path in **d**, showing that the emission of one dislocation can change the strain field near the crack and thus turn the crack direction. Scale bar, 2 nm. (**e**) The atom displacement mapping between 0 and 10 s. All the unit cell locations in monolayer MoS_2_ are determined by Gaussian fitting of the TEM images in **a**,**b** and then the displacement vector of each unit cell between 0 and 10 s can be calculated by subtraction. The displacement vectors are presented directly in **e**, and the colour stands for the displacement magnitude. Relative slip between two planes and creation of one dislocation can be seen clearly inside the red box. There are several surface atoms lost during cracking.

The dislocation dynamics near the crack tip was investigated by more *in situ* TEM observations. For example, in Fig. 3a, the crystallinity is perfect near the crack tip (including areas 1, 2 and 3). After 10 s, the crack propagates to area 2 and there is a new dislocation in area 3 (Fig. 3b). The GPA strain analysis for the image at 10 s shows that the maximum tensile strain (ɛ_22_) direction is towards the newly emitted dislocation (Fig. 3c). After 90 s, the crack passed through areas 2 and 3 and arrived at area 4 (Fig. 3d). By identifying the positions for all the unit cells in Fig. 3a,b, we can plot the atomic displacement map with vectors (Fig. 3e). Figure 3e shows the atomic model for the emission of a dislocation on the surface near the crack tip. The red box highlights the dislocation emission zone. It can be easily seen that the slip plane connecting the crack surface and the dislocation core is (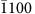) plane. After the emission of this dislocation by gliding toward one side of the crack (right side here), the axial strain field at the crack tip (Fig. 3c) is different from the strain field when one dislocation stays in front of the crack path (Fig. 2e,f). The mode I tensile strain (ɛ_11_) for the original cracking direction becomes lower, whereas tensile strain (ɛ_22_) is higher and the direction from crack tip to the dislocation has the maximum ɛ_22_, and therefore the crack turns direction in area 2. Except for the dependence of crack propagation paths on the dislocation emissions as we observed here, other factors including external stress and surface adsorbents can also play a role to determine the crack path.

As mentioned before, the surface absorbents function to stabilize the cracking process that makes it possible for *in situ* observations. We performed *in situ* cracking experiments in the pristine sample, as well as the sample after low-intensity beam shower without beam damage (defect analysis shown in Supplementary Table 1) for 10 and 25 min, respectively. Supplementary Figure 8 presents three cases of cracking by strain maps corresponding to the three conditions. The amount of absorbents on the MoS_2_ surface is reduced by increasing time of beam shower, confirmed by the direct TEM observations (Supplementary Figure 7). Mode I tensile strain (ɛ_22_) decreases with less amount of absorbents (Supplementary Fig. 8), in agreement to the greater stabilization effect by more amount of absorbents. The statistics on the cracking speed and emitted dislocation density per unit length of crack is presented in Supplementary Table 2. Smaller absorbents on the sample generate larger number of dislocations. The reason for this is that larger strain at the crack tip and slower cracking speed for the sample with more absorbents allows the dislocations at the crack tip to climb with the propagating crack (supplementary Figure 8). Meanwhile, with smaller absorbents, the newly emitted dislocations have less time to move and dislocations will be caught up by the propagating crack and escape from the surface, and then another new dislocation is emitted in the following cracking process (Supplementary Fig. 8). The local strain change caused by the removal of adsorbents may influence the cracking path. However, the ability of emitting dislocations is intrinsic for MoS_2_, and the emitted dislocations can significantly change the stress field at the crack tip and affect the cracking path prominently (Fig. 3).

According to our observations, the blunting of the crack tip with a radius of up to 2 nm in MoS_2_ can be identified during cracking (Supplementary Fig. 9) in line with the emission of a large density of dislocations. The maximum dislocation density occurs right at the forefront of the crack tip, whereas the post-cracking part has fewer dislocations, partially because of the released dislocations after cracking, leaving steps and corrugations. The material constant, the size of the plastic zone (*r*_c_) at the crack tip, can be estimated by models or simulations^26^; 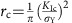 for the plane stress condition, where *σ*_Y_ is the yield stress. For monolayer MoS_2_, the observed *r*_c_ is equal to 2–5 nm. Linear fracture mechanics can be applied if the cracking is brittle and *r*_*c*_<<*a*, where *a* is the characteristic dimension of intrinsic defects in the object. However, near the cracks our monolayer MoS_2_ sample has only sulfur vacancy defects, implying that *a* approaches 0 nm. This implies a higher probability of generating plasticity.

### Environmental assistance of cracking in 2D material

TMD materials have been known to be susceptible to environment^18^,^27^. In the following we will discuss about the environment-assisted cracking of monolayer MoS_2_, the so-called stress corrosion crack (SCC). For SCC, the *K*_IC_ usually becomes smaller because of a chemical reaction assisted by release of the decohesion energy. Meanwhile, the yield stress also decreases. The as-grown monolayer MoS_2_ sample on a sapphire substrate was exposed to ultraviolet (UV) light with controlled humidity inside the chamber (see Methods)^27^. Highly oxidative chemical groups, such as O_3_ and oxygen-related radicals, can be created under UV that can quickly react with MoS_2_. This method was first applied to visualize the grain boundaries of 2D materials^28^.

**Figure 4:**
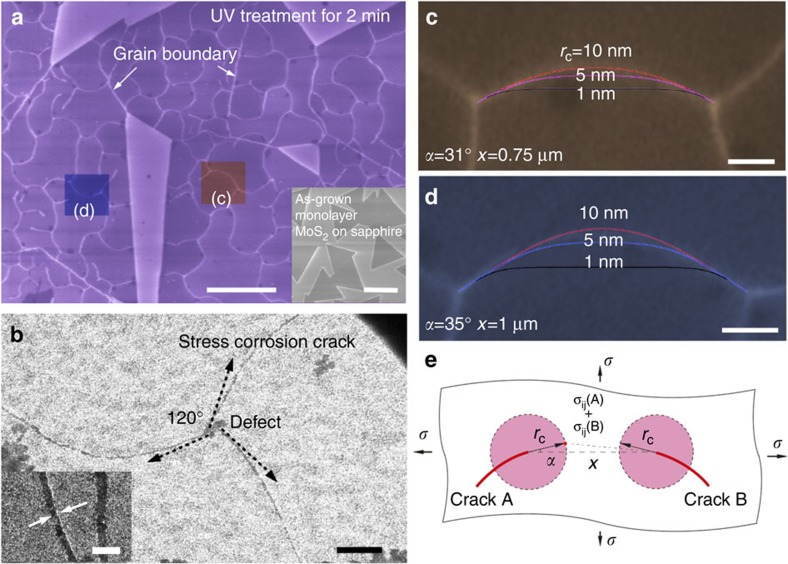
**Stress corrosion cracking of monolayer MoS**_**2**_. (**a**) Scanning electron microscope image of crack pattern generated after 2 min of UV light exposure under humid conditions on MoS_2_. Scale bar, 2 μm. Inset shows an image of the as-grown sample. Scale bar, 5 μm. (**b**) TEM dark-field image of triple crack paths initialized from one defect similar to (**a**). Scale bar, 100 nm. Inset highlights the crack. Scale bar, 20 nm. (**c**,**d**) Magnified images of two areas in **a**, two cracks meet in the centre and are curved by each other because of the strain field. The three theoretically simulated paths using linear elasticity theory with different *r*_c_ are fitted with the experimental paths. Scale bar (**c**), 150 nm. Scale bar (**d**), 200 nm. (**e**) Schematic showing the stress state of two meeting cracks.

The scanning electron microscopy image of a MoS_2_ sample after 2 min of UV exposure is shown in Fig. 4a with that of the pristine sample in the inset. A high density of crack patterns was observed. The MoS_2_ monolayer is subject to a biaxial in-plane stress mainly caused by the robust adhesion between MoS_2_ and the sapphire substrate^29^. The grain boundaries between the triangular-shaped MoS_2_ single crystals can first be determined by the rather straight lines (Fig. 4a). A zoom-in DF-TEM image (Fig. 4b) clearly reveals the triply branched cracking, stemming from some point defects (∼50 nm size). The angle between crack directions is 120° and the initial directions of cracks are all along the [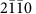] (zigzag) direction (Fig. 4b), as determined by electron diffraction (see Supplementary Fig. 10) that is the same as the cracking plane in the pristine sample. The ageing effect after cracking can be excluded, because the crack retains a uniform width. We also note that the cracks propagate in a random manner in another MoS_2_ sample subject to the same UV treatment conditions after being transferred onto the TEM grid (Supplementary Fig. 11). Instead, in pristine MoS_2_ sample on sapphire, the cracks interact with each other because of the elastic field induced by MoS_2_–sapphire interactions. Because the monolayer MoS_2_ is ultrathin, all the cracks can be treated as steady-state (low strain rate) semi-infinite crack propagation in linear elasticity theory^30^. Therefore, the triply branched cracks have to turn their direction clockwise (or counterclockwise) coherently to satisfy the elastic stress/strain equilibrium (Fig. 4b).

By applying a linear elastic field under a biaxial stress condition in classical fracture mechanics and assuming that the stress field (out of plastic zone) extends to a distance *r*_c_ from the crack tip, we can simulate the crack path between two cracks that approach to each other (see Supplementary Note 1). The shape of the crack path does not depend on the far-field stress but relies on *r*_c_. Figure 4c,d shows two examples of cracks approaching and finally merging with different initial conditions (tip spacing *x* and cracking angle *α*, Fig. 4e). The experimental images are overlaid with the cracking paths simulated with different *r*_c_. More brittle materials (smaller *r*_c_) can turn the direction of cracking easily when affected by other elastic fields (the scheme can be found in Fig. 4e). The extreme case for *r*_c_=0 will be a straight line. The experimental cracking paths (Fig. 4c,d) are located between *r*_c_=5 nm and *r*_c_=10 nm.

On the other hand, the post SCC cracking MoS_2_ sample is rechecked by high-resolution scanning transmission electron microscope (STEM) and electron energy loss spectra (EELS) (Supplementary Fig. 12). The pre-edge background subtracted EELS spectra shows that the cracking under UV induces stoichiometric deficiencies for sulfur near the crack (∼5 nm from the crack surface/edge) as compared with the region away from the crack. The large local strains near the crack tip will elevate the chemical reaction speed and thus lead to higher defect concentrations near the crack tip. Density functional calculations reported that the dislocations dynamics can be much easier in defective MoS_2_ (ref. 31). Consequently, the greater sulfur deficiencies will increase the plasticity (emission and dynamics of dislocations). By EELS line scan and STEM images, we confirmed the width (dimension) of this higher deficiency zone near crack under UV environment attack is ∼5–8 nm, in congruent with the continuum mechanics simulation results. Therefore, by continuum mechanics modelling and EELS we conclude that the tip plastic zone for SCC of MoS_2_ is 5–10 nm. This value is larger than that associated with pure cracking (2–5 nm). However, this is not contradictory to the usual plasticity enhancement of ceramic materials under corrosive environment^32^.

In concluding, we note that for a long time researches have been interested in determining the exact atomic structure at the plastic crack tip zone^6^ that is a singular field according to classical fracture theory^3^. Observation at the crack tip is the most direct approach but has been limited by both materials and instruments. Apart from providing an atomistic picture of the crack tip, here we have observed the emission and dynamics of dislocations in MoS_2_ sample with 1% sulfur vacancy concentration. In addition, the 2D mode of cracking in MoS_2_ has, to our knowledge, not been reported before and has broader conceptual significance. We observed the edge dislocation emission that is different from the more frequently observed screw dislocation emissions in 3D cracks^33^. In 2D cracking, there is easier fluctuation in 3D space, and hence the extreme singular strain at the crack tip can be relaxed in part by 3D fluctuations. There is a higher atomic diffusion rate at the surface, and hence there could be easier dislocation climb in 2D. Such processes may account for the higher ductility/plasticity in 2D materials when compared with their 3D bulk counterparts. The plastic zone of MoS_2_, which is a few nanometers in size, may be enlarged or shrunk by temperature control or defect/dopant engineering for various purposes.

## Methods

### MoS_2_ grown on sapphire by chemical vapour deposition

MoS_2_ film was synthesized following a previous report^20^. At first, 0.3 g MoO_3_ powder was placed in a ceramic boat located in the centre heating zone of the furnace and S powder was placed in a separate ceramic boat at the upper stream side maintained at 150 °C during the reaction. An Ar carrier gas (70 sccm, chamber pressure=40 Torr) brought the S and MoO_3_ vapours to the target sapphire substrate that was located at the downstream side. The centre heating zone was heated to 635 °C at a ramping rate of 15 °C per min. After reaching 635 °C, the heating zone was maintained there for 30 min and the furnace was then naturally cooled down to room temperature.

### Transfer of MoS_2_ on sapphire to TEM grid

PMMA (A4, Chem) was spin-coated onto the as-grown MoS_2_ (2,000 r.p.m., 1 min) as a supporter and a protective layer with no post-annealing step. The MoS_2_ and PMMA support were then detached from the sapphire substrate by floating the PMMA/MoS_2_/sapphire, with the PMMA side up, in a hot 2 M NaOH solution for 10 min. The PMMA/MoS_2_ was then transferred into deionized water (4 times) for removing any remaining NaOH in the sample. Finally, the PMMA/MoS_2_ was scooped out in pieces and placed on a Quantifoil TEM grid with a gold-supported thin film (PELCO, 200 mesh, gold, 1.2 μm holes). The grids were kept in an ambient oven at 80 °C for 1 min to increase adhesion between the PMMA/MoS_2_ and the grid. PMMA was ultimately removed by acetone vapour. Before each TEM measurement, the grids were annealed at 180 °C in a vacuum chamber (∼7.5 × 10^−5^ Torr) for at least 12 h.

### *In situ* TEM

TEM observations were conducted using a JEM ARM 200F operated at 80 kV that is lower than the knock-on damage threshold for monolayer MoS_2_ (90 kV)^34^. Acquisition time for bright-field and DF images was 1 s. DF image was obtained using the smallest objective lens aperture. The HRTEM imaging acquisition time was 0.32–1 s. The Fourier filtering method was applied to reduce the noise level and enhance contrast of the images^22^. The beam intensity was <0.01 pA nm^−2^ in order to obtain the HRTEM images. The beam intensity at the sample in TEM mode is measured by the screen current multiplied by the square of magnifications. Because of contamination problems, it was difficult to obtain high-resolution STEM images during or after cracking. Annular dark-field STEM imaging was conducted using a probe aberration-corrected JEM ARM200F operated at 80 kV. Annular dark-field images were acquired at a 20 mrad convergence angle, with beam current 120–150 pA, beam size ∼1 Å, acquisition times of 40 μs per pixel and collection angle from 50 to 180 mrad. Because the energy of the low-voltage electron beam was below the damage threshold energy, the pristine MoS_2_ remains stable after regular scanning.

### UV light exposure

MoS_2_ grown on sapphire was placed in a chamber equipped with a low-pressure Hg lamp (LH-arc, Lichtzen, with an output of 20 mW cm^−2^), with the majority of emitted light at a wavelength of 254 nm and ∼10% of emitted light at a wavelength of 185 nm. Humidity was introduced into the chamber by connecting it to a humidifier. The humidity level in the chamber was monitored using a hydro-thermometer (accuracy of ±3%). The humidifier was disconnected from the chamber after reaching 65% humidity level. The MoS_2_ on sapphire was then irradiated under UV light for 2 min. Radicals are generated under UV and humidity conditions as follows: O_2_→O_3_; O_3_+H_2_O→O_2_+H_2_O_2_; 2O_3_+H_2_O_2_→2OH*+3O_2_; H_2_O→H*+OH*.

### Scanning electron microscopy

The surface morphology of the TMDs on the SiO_2_/Si substrate was examined by field-emission SEM (JSM7000F, JEOL, Japan). An accelerating voltage of 15 kV was utilized to obtain a high contrast at different magnifications.

### Data availability

The data that support the findings of this study are available from the corresponding author on reasonable request.

## Additional information

**How to cite this article**: Ly, T. H. *et al*. Dynamical observations on the crack tip zone and stress corrosion of two-dimensional MoS_2_. *Nat. Commun.* **8**, 14116 doi: 10.1038/ncomms14116 (2017).

**Publisher's note**: Springer Nature remains neutral with regard to jurisdictional claims in published maps and institutional affiliations.

## Supplementary Material

Supplementary Information Supplementary Figures, Supplementary Tables, Supplementary Notes and Supplementary References
