# Supplementary material for: Dynamical observations on the crack tip zone and stress corrosion of two-dimensional MoS2
Source: Nat Commun. 2017 Jan 18;8:14116. doi: 10.1038/ncomms14116 (PMC5253633; doi:10.1038/ncomms14116)
Supplement: Supplementary Information — Supplementary Figures, Supplementary Tables, Supplementary Notes and Supplementary References [file ncomms14116-s1.pdf]

Supplementary Figures

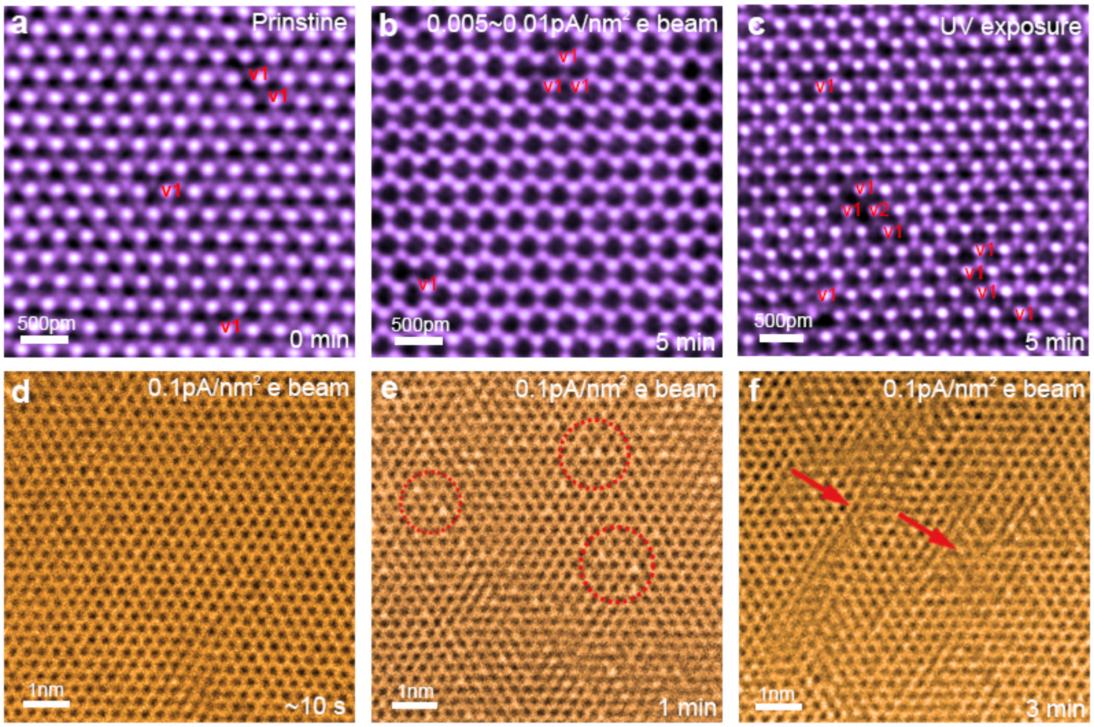

Supplementary Figure 1| a-c, Typical STEM-HAADF images for the pristine monolayer MoS_2 sample, sample after low-density electron-beam irradiation for 10 min and sample after UV exposure for 5 min. The single and double sulphur vacancies are highlighted by v1 and v2, respectively. The HAADF images are taken in different conditions so that the contrast of Mo and S atoms is not fixed. **d-f**, TEM image series for the monolayer MoS_2 sample after high density electron beam irradiation for 10 s, 1 min and 3 min, respectively. Some sulphur vacancies are labeled in **e**, and extended line defects are highlighted by red arrows in **f**. The beam dose labeled in the images show the pre-treatment beam shower conditions on the samples, not the conditions for STEM/TEM imaging.

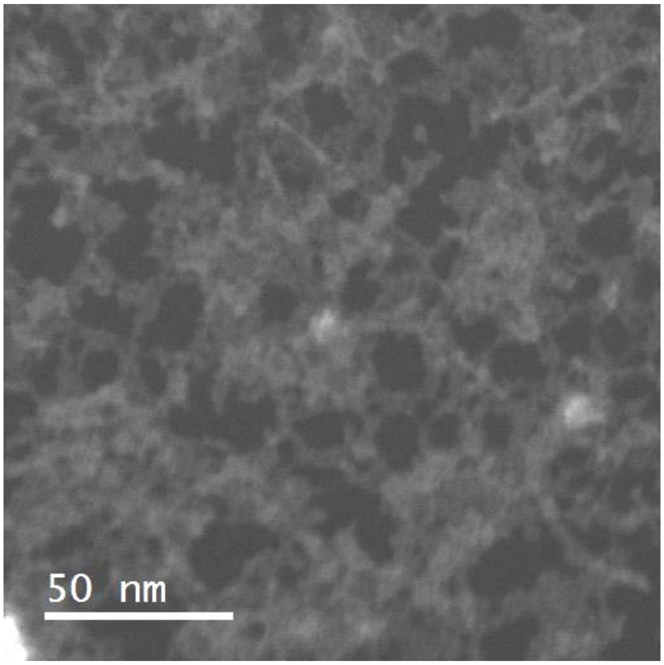

Supplementary Figure 2| The residues absorbed on the surface of the as-prepared MoS_2 TEM sample imaged by STEM ADF technique.

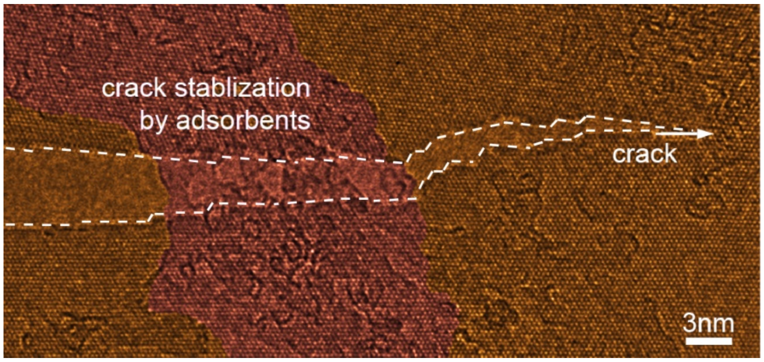

Supplementary Figure 3 | TEM image for a crack stabilized by absorbents bridging the two opposite edges in the post-crack part. The adsorbents are highlighted in red.

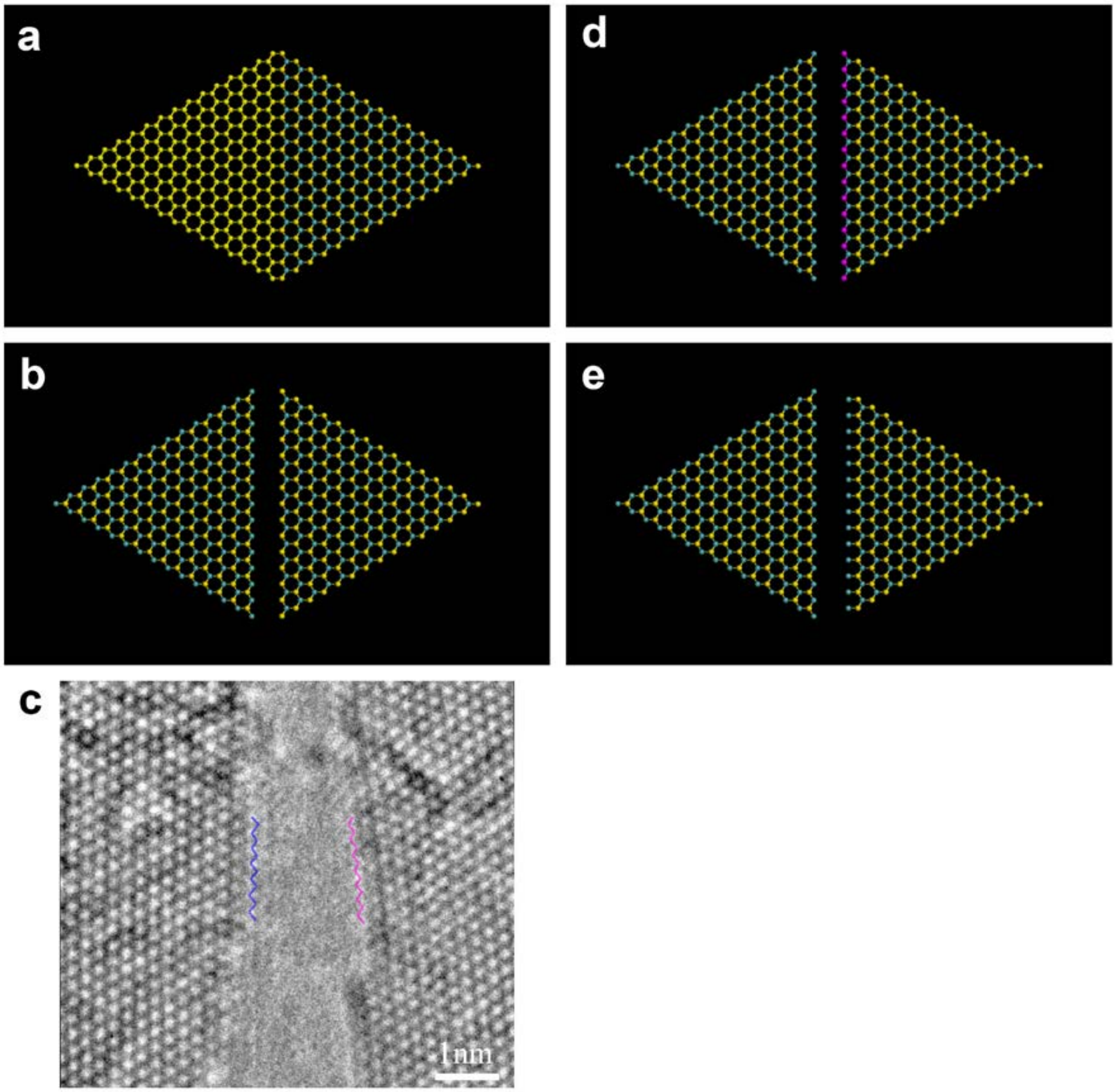

Supplementary Figure 4| (a) Atomic structure of perfect MoS_2. (b) Cleavage along the zigzag plane $(01\bar{1}0)$. (c) HRTEM after cracking in which the S-Mo-S zigzag edge (purple) and Mo-S-Mo zigzag edge (pink) are highlighted. (d) The S vacancies can exist in the MoS_2 edge, in particular by irradiation damage. (e) After cracking, the outmost S layer is lost and Mo is left with many dangling bonds at one of the edges. The experimental TEM image of the MoS_2 crack after great e beam damage can be found in ref (34) of the main text.

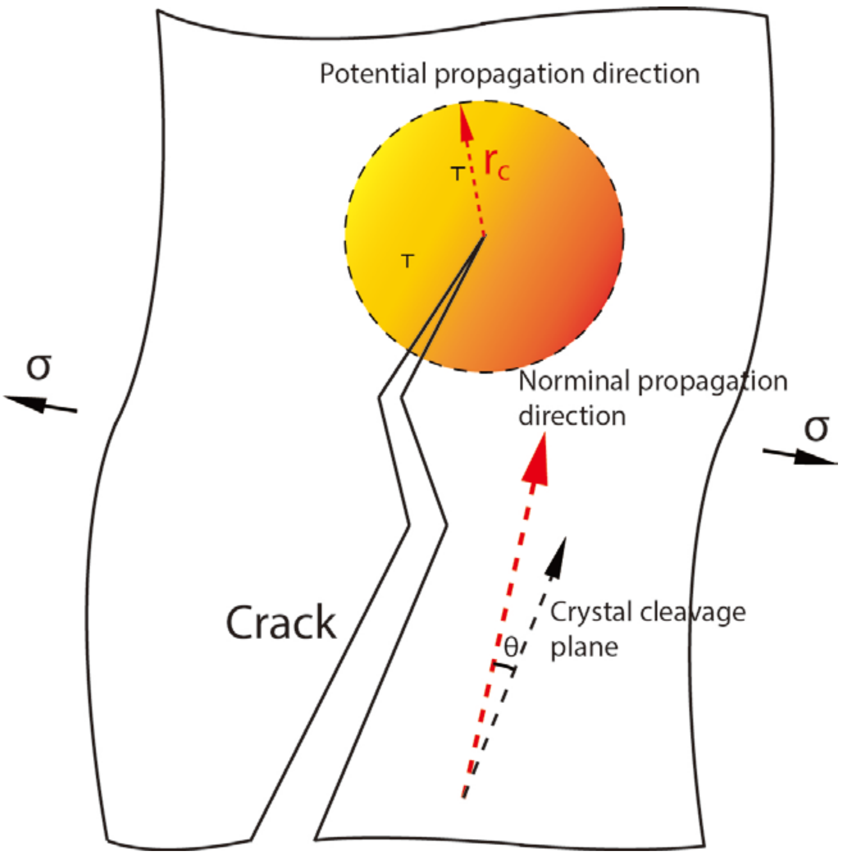

Supplementary Figure 5| Scheme showing the crack. r_c is the radius of the plastic zone, θ is the angle between the cleavage plane and the nominal crack direction, and σ is the far-field stress.

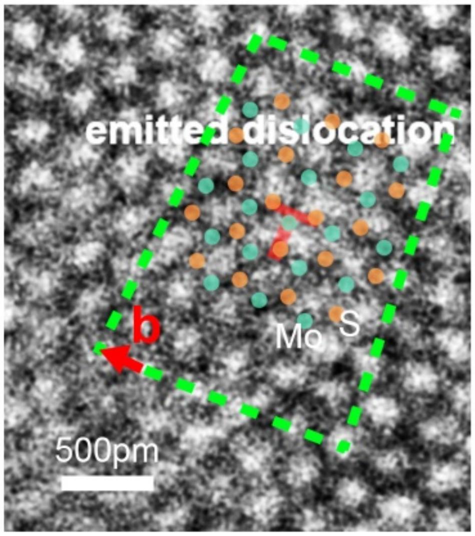

Supplementary Figure 6| HRTEM image of the dislocation zone near crack tip with Mo (green) and S (yellow) atoms identified.

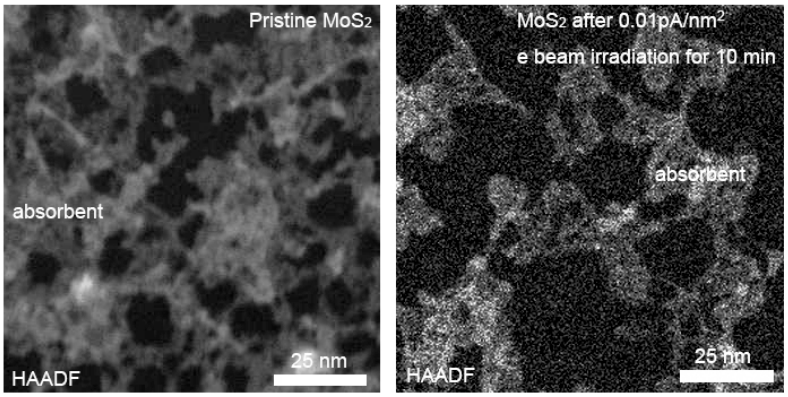

Supplementary Figure 7| STEM-HAADF images for pristine MoS_2 and MoS_2 sample after 0.01 pA/nm^2 e-beam irradiation for 10 min, demonstrating the absorbent can be evaporated gradually by such low beam current electron beam shower.

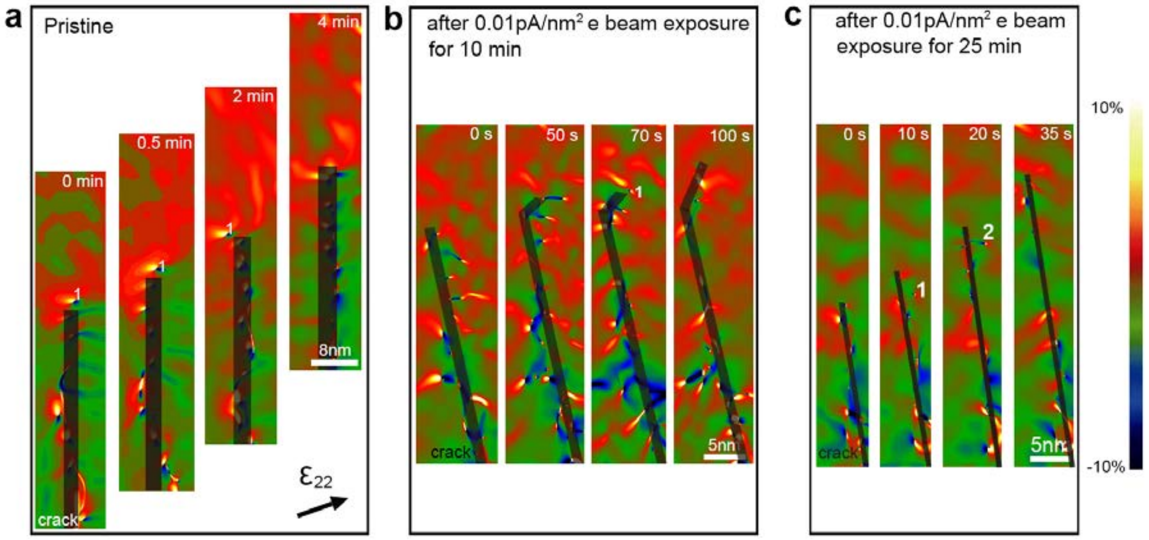

Supplementary Figure 8| a-c, Three examples of *in situ* TEM image series (GPA strain analysis on ε_{22}) on the cracking process in the same monolayer MoS_2, after e-beam exposure at different times of low intensity (0.01 pA/nm^2) (which will not create defects rapidly). All the *in situ* image series are spatially aligned. Newly emitted dislocations are labelled by numbers. The emitted dislocation density per unit length of crack is shown in Supplementary Table S2. It should be noted that the strain analysis by GPA method may introduce error (20% overestimation) in particular in the extreme strain fields (±10% strain zone) in the vicinity of defects (i.e. 2 nm radius region around dislocations) due to corrugations or buckling of the atomic layer, while the small strain fields in flat parts will not be affected.

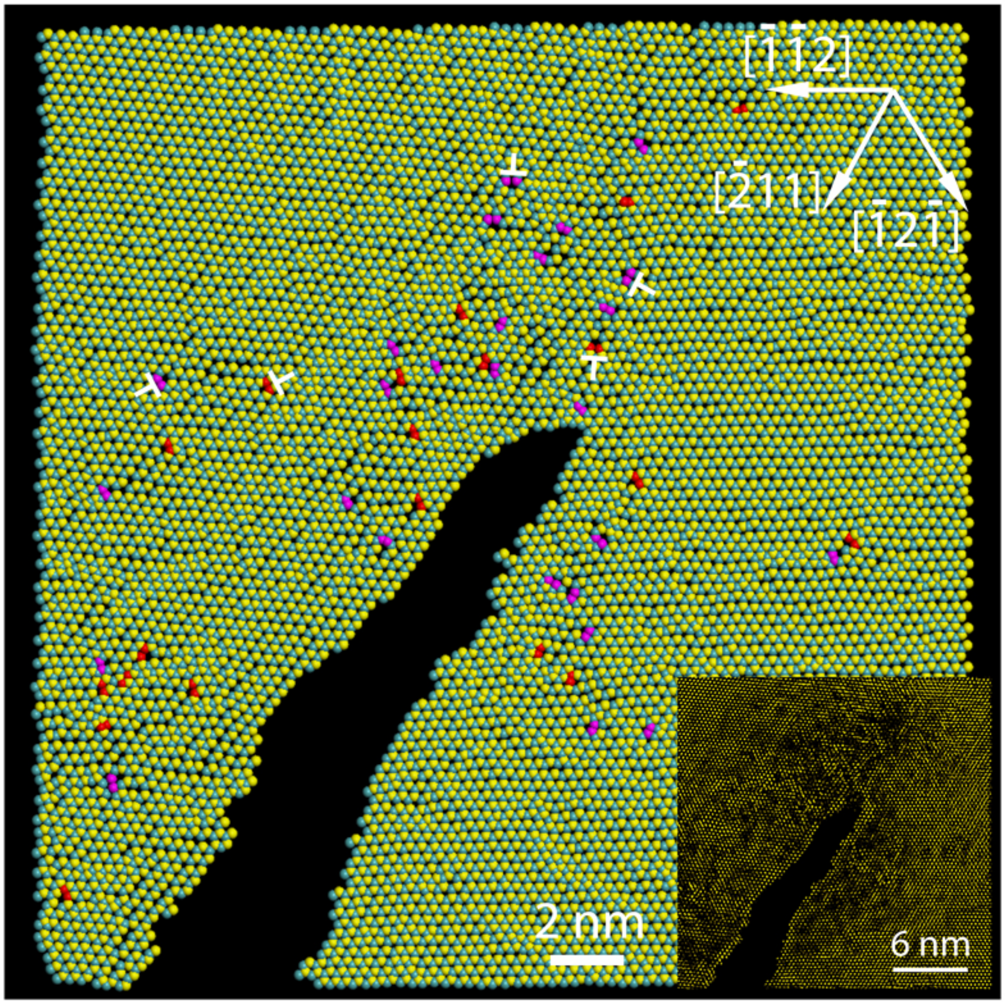

Supplementary Figure 9| Reconstructed atomic structure of the one crack tip zone during crack obtained from high-resolution TEM image. The green and yellow balls represent Mo and S(2) atoms, while the red and violet balls highlight the Mo and S(2) atoms at all dislocation cores. Inset is the filtered HRTEM image of the forefront of the crack.

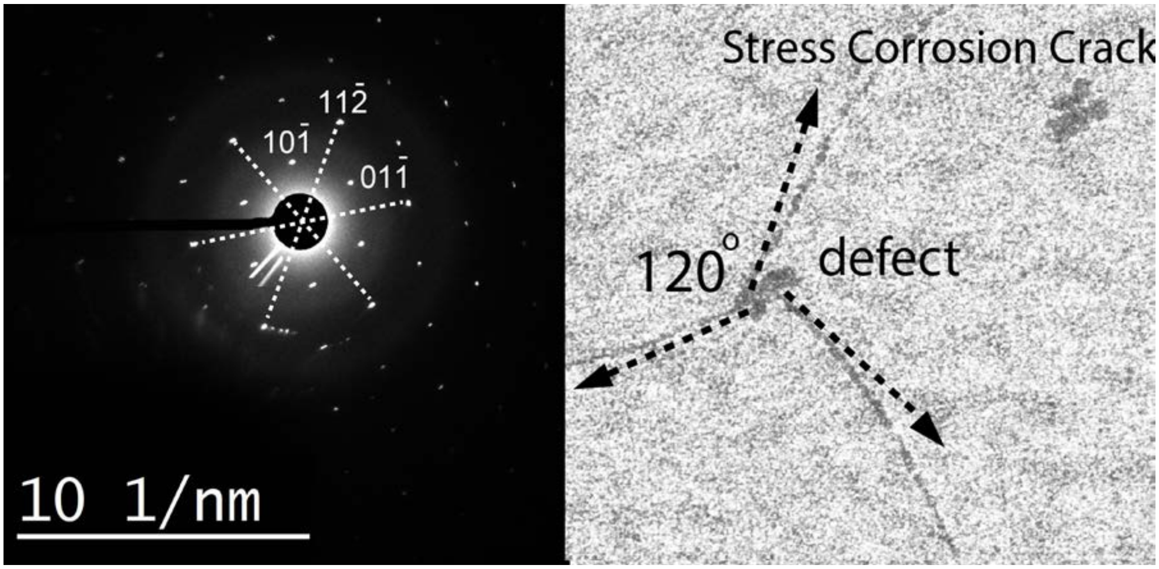

Supplementary Figure 10| The electron diffraction pattern (left) revealing that the SCC path (right) is initialized along the $[11\bar{2}0]$ crystal direction, the same as the cracking plane without the corrosive condition. The last index in four index method are skipped in the diffraction pattern (left).

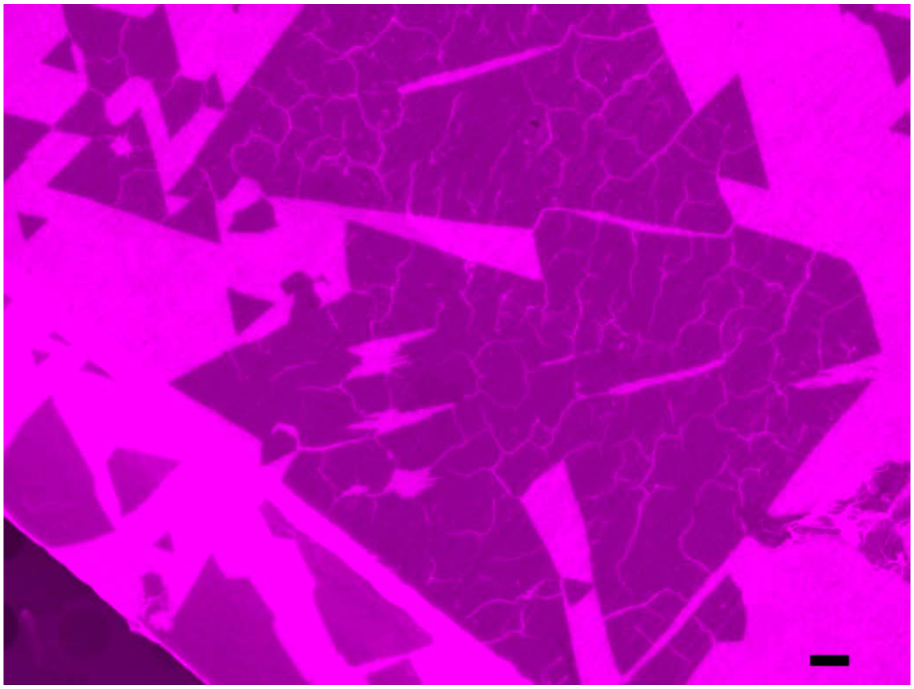

Supplementary Figure 11| The MoS_2 monolayer transferred onto a TEM grid after growth (not on sapphire substrate). After this, the UV-assisted cracking exhibits a random manner, different from the one on the sapphire substrate shown in the main text. The stress in this case is much smaller or even totally released after transfer in some region, in contrast with the much greater stress for MoS_2 directly grown on sapphire substrate. Scale bar is 1 μm.

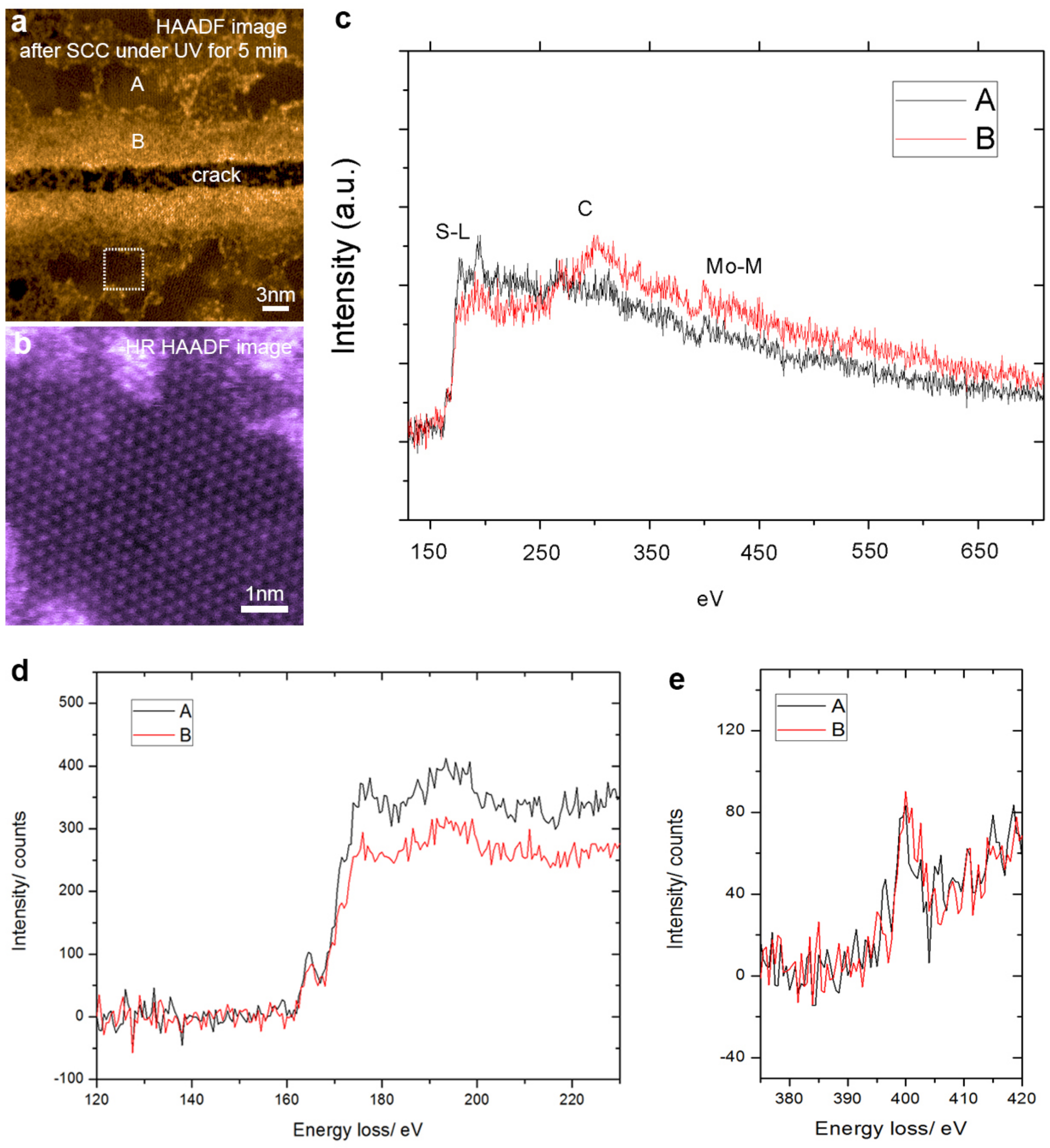

Supplementary Figure 12| **a**, HAADF image of one crack area in MoS_2 monolayer sample after SCC under UV exposure for 5 min, UV conditions are the same as in main text Figure 4. **b**, Magnified HAADF image for the white box in a. **c**, EELS spectrum (pre-edge background subtracted for Sulphur L edge) for the two areas “A” and “B” in marked in **a**. A and B EELS are extracted from a single line scan. **d**,**e**, The EELS spectra of S L edge and Mo M edges at A and B positions, integrated and averaged ten single spectrum respectively from one line scan. We use the experimentally measured spectrum between 120-150 eV range and 360-390 eV range for pre-edge background subtraction for the S L

edge and Mo M edge, in order to exclude the effect of the plasmon peaks (peak position at 50 eV by our EELS measurement) from the carbon residues. After pre-edge background subtraction, the EEL spectra converge to zero before the desired edges. By using same condition to obtain the EELS from A and B areas, we can see from **d** and **e** that the S edge at the A is considerably higher than B while Mo edge keeps similar, manifesting the higher S deficiencies near the cracks after SCC process.

Supplementary Table 1. Statistics on the defects in MoS_2 under different conditions.

Sample	S single vacancy (V_{S1})	S double vacancy (V_{S2})	Mo single vacancy (V_{Mo1})	Dislocation	Area counted(nm^2)
**Pristine MoS_2 fabricated by CVD**					*(by HAADF images)*
1	2.3%	0.7%	0%	0%	30
2	2.6%	0.4%	0%	0%	50
3	1%	0%	0%	0%	50
**Average**	**1.9±0.85%**	**0.36±0.35%**	**0%**	**0%**	
**MoS_2 after 0.005-0.01pA/nm^2 electron beam irradiation for 5-15 min** *(by HAADF)*					
1	4%	1%	0.1%	0%	100
2	3.5%	1.2%	0%	0%	100
3	1%	0.4%	0.1%	0%	50
4	3.3%	1%	0%	0%	100
5	2.3%	0.1%	0%	0%	100
6	1.9%	0.2%	0%	0%	100
7	1%	0.6%	0.2%	0%	100
8	0.4%	0.1%	0%	0%	250
9	0.8%	0.4%	0.2%	0%	50
10	1.2%	0.4%	0%	0%	50
**Average**	**1.9±1.2%**	**0.54±0.39%**	**0.04%**	**0%**	
**MoS_2 after 0.1pA/nm^2 electron beam irradiation**					*(by HRTEM images)*
1(pristine)	1.3%		0%	0%	500
2(30s)	3.4%		0%	0%	250
3(1min)	4%		0%	0%	250
4(3min)	Line defects emerge		0.4%	0%	250
**MoS_2 after UV environment exposure for 5 min**					*(by HAADF images)*
1	5.5%	0.3%	0%	0%	32
2	4%	0.2%	0%	0%	50
3	8.2%	0%	0%	0%	32
4	4.4%	0.5%	0%	0%	75
**Average**	**5.5±1.9%**	**0.3±0.2%**	**0%**	**0%**	

Supplementary Table 2. Statistics on the cracking process in the same MoS_2 sample.

	Pristine MoS_2	After 0.01 pA/nm^2 e-beam exposure for 10 min	After 0.01 pA/nm^2 e-beam exposure for 25 min
Emitted dislocation density per unit length (nm^{-1})	**0.03**	**0.1**	**0.15**
Cracking speed (nm/s)	**0.13**	**0.15**	**0.33**

Supplementary Note 1

The crack path is simulated using two basic assumptions, steady state cracking and the thin film assumption, which can be applied to a monolayer here. We simulated the crack in the mixed mode loading using K_{II}=0 criterion[1,2]. The following stress distribution for a semi-infinite crack is applied[2], $\sigma_{xx} = \frac{K_I}{\sqrt{2\pi r}}\cos\frac{\theta}{2}(1\text{-}\sin\frac{\theta}{2}\sin\frac{3\theta}{2})$, $\sigma_{yy} = \frac{K_I}{\sqrt{2\pi r}}\cos\frac{\theta}{2}(1 + sin\frac{\theta}{2}sin\frac{3\theta}{2})$, $\sigma_{xy} = \frac{K_I}{\sqrt{2\pi r}}\cos\frac{\theta}{2}sin\frac{\theta}{2}cos\frac{3\theta}{2}$, where K_I is the mode I stress intensity factor, r is the distance to the crack tip, and $\theta$is the angle with respect to the crack surface. The stress field at the edge of the plastic zone around tip A is considered as a stress field arising from crack A with small perturbations of the stress field of crack B and vice versa. The crack propagation rate is proportional to K_I, and in our simulations, we assume that K_I is constant, which is reasonable because the entire MoS_2 crystal is homogeneously strained by the underlying sapphire substrate. During each simulation step, the stress field out of the crack tip plastic zone is calculated and the K_{II}=0 criterion is applied to determine the next step cracking direction. The size of plastic zone r_c is the only variable in our simulations. All the simulations are developed by home-built Matlab codes.

Supplementary References.

1. B. Cotterell, J.R. Rice, *Int. J. Fract.***16**, 155–169 (1980).

2. C.-T. Sun, Z.-H. Jin, *Fracture Mechanics*, Elsevier (2012)
